# Respiration-Driven Brain Oscillations in Emotional Cognition

**DOI:** 10.3389/fncir.2021.761812

**Published:** 2021-10-27

**Authors:** Shani Folschweiller, Jonas-Frederic Sauer

**Affiliations:** ^1^Institute for Physiology I, University of Freiburg, Freiburg, Germany; ^2^Faculty of Biology, University of Freiburg, Freiburg, Germany

**Keywords:** network, emotion, respiration, oscillations, embodied cognition, neuronal synchronization, neuronal circuits, slow oscillation

## Abstract

Respiration paces brain oscillations and the firing of individual neurons, revealing a profound impact of rhythmic breathing on brain activity. Intriguingly, respiration-driven entrainment of neural activity occurs in a variety of cortical areas, including those involved in higher cognitive functions such as associative neocortical regions and the hippocampus. Here we review recent findings of respiration-entrained brain activity with a particular focus on emotional cognition. We summarize studies from different brain areas involved in emotional behavior such as fear, despair, and motivation, and compile findings of respiration-driven activities across species. Furthermore, we discuss the proposed cellular and network mechanisms by which cortical circuits are entrained by respiration. The emerging synthesis from a large body of literature suggests that the impact of respiration on brain function is widespread across the brain and highly relevant for distinct cognitive functions. These intricate links between respiration and cognitive processes call for mechanistic studies of the role of rhythmic breathing as a timing signal for brain activity.

## Introduction

**Figure 1:**
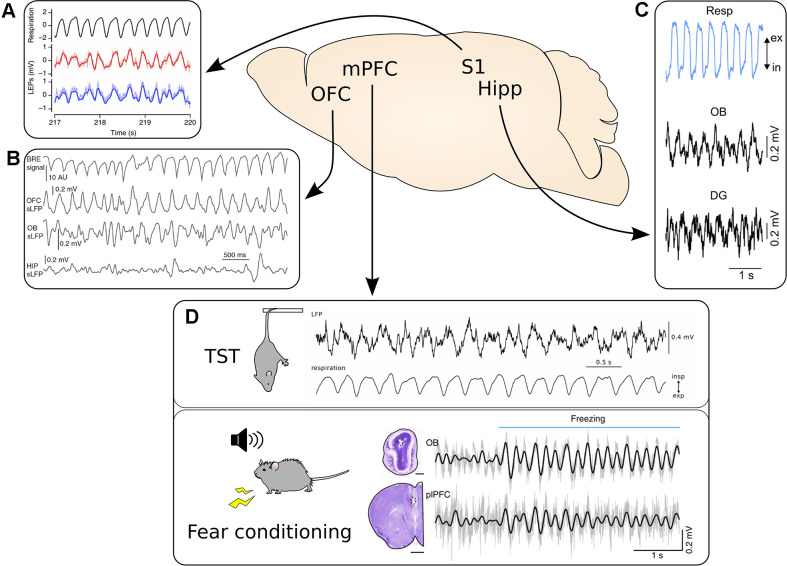
Respiration-driven slow oscillations are widespread in the cortex. **(A)** Top, black: respiratory trace measured with a thermistor. Bottom, red/blue: LFP traces from two recording sites located 300 μm apart in the whisker barrel cortex during accelerated breathing induced by exposure to hypoxic air. Reprinted from Ito et al. (2014) under CC BY 4.0. **(B)** Video-based measuring of breathing (BRE signal, top) and simultaneous LFP recordings from OFC, OB, and hippocampus (HIP) during immobility. Reprinted from Kőszeghy et al. (2018) under CC BY 4.0. **(C)** Top, blue: respiration trace measured with a thermocouple. Middle and bottom, black: simultaneous LFP recordings from the OB and dentate gyrus (DG) of the hippocampus. Reprinted from Nguyen Chi et al. (2016) under CC BY 4.0. **(D)** The mPFC shows strong entrainment by respiration across a variety of behavioral states. Top: mPFC LFP recorded during immobility during the tail suspension test. Bottom: The simultaneous respiratory trace measured with a thermocouple. Adapted from Biskamp et al. (2017) under CC BY 4.0. Bottom: Synchronous LFP signal from the OB and prelimbic cortex (plPFC) recorded during auditory fear conditioning. Please note the increase in the amplitude of the 4 Hz rhythm in both LFP signals at freezing onset, indicated by the horizontal blue bar. Adapted from Moberly et al. (2018) under CC BY 4.0. *p < 0.05.

Breathing ensures the constant supply of oxygen to the organism while eliminating CO_2_ that is produced during metabolic processes. The perpetual sequence of inspiration and expiration is generated by an intricate network of excitatory and inhibitory cells in the brainstem, in particular in the ventral respiratory group (see Feldman and Del Negro, 2006 for a review on the network mechanisms underlying the generation of the breathing rhythm). However, viewing breathing as a one-way process going out of the central nervous system (CNS) gives an incomplete picture. Rather, rhythmic breathing exerts a backward influence on the brain in form of rhythmically entrained brain oscillations. More than six decades ago, Adrian found in seminal recordings from hedgehogs that the olfactory bulbs (OBs), the first relay station of the olfactory pathway, show respiration-synchronous oscillations in the local field potential (LFP, Adrian, 1942). Further experiments revealed that these oscillations are propagated to olfactory areas such as the piriform cortex, giving rise to the hypothesis that respiration-synchronous oscillations might aid the processing of olfactory inputs (Fontanini and Bower, 2006). In recent years, an increasing number of studies additionally reported respiration-synchronous brain oscillations in various brain regions, including higher-order areas involved in cognitive functions (Figure 1; Ito et al., 2014; Lockmann et al., 2016; Nguyen Chi et al., 2016; Biskamp et al., 2017; Zhong et al., 2017; Karalis and Sirota, 2018; Moberly et al., 2018; Bagur et al., 2021). These data suggest that respiration-related oscillations [also called respiration rhythms (RRs)] might fulfill general functions in neuronal circuits that extend beyond the processing of olfactory inputs. In this review, we will discuss the relevance of RR for emotional cognition. We will start with the mechanism(s) giving rise to RR in the forebrain, describe where RR is found in networks involved in emotions, and focus then on the implications of these oscillations for cognitive processes. We will conclude with the most important points onto which further light should be shed.

## Mechanisms of Generation of Respiration-Driven Brain Oscillations

Building on the observations by Adrian, unit recording revealed that neurons in the OB are biased in their spiking by nasal airflow, irrespective of whether or not specific olfactory stimuli are presented (Macrides and Chorover, 1972). Calcium imaging from the axons of olfactory sensory neurons (OSNs) impinging on mitral cells, the main output neuron in the OB, furthermore showed that presynaptic activity is synchronized with nasal breathing, indicating that OSNs are receptive to air movement and transmit the air flow-induced activity to the OB (Carey et al., 2009). Direct recordings from OSNs indeed demonstrated that these neurons not only respond to specific odorants but are also mechanosensitive (Grosmaitre et al., 2007). Individual OSNs detect odorants through the expression of specific G-protein-coupled odorant receptors (Buck and Axel, 1991). Interestingly, the mechanosensitive properties of OSNs rely on the odorant receptors themselves (Connelly et al., 2015), with some but not all odorant receptor types being sensitive to nasal air movement. These data suggest that the RR activity in the OB is driven by airflow-induced spikes in a subset of OSNs.

**Figure 2:**
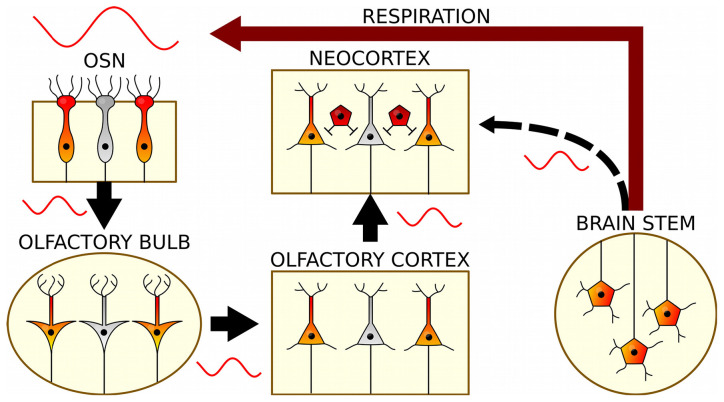
The generation and propagation of respiration-related oscillations in the brain. Rhythmic breathing is generated in the brain stem and feeds back onto neocortical and hippocampal circuits *via* nasal airflow (red): Air movement through the nasal cavity excites OSNs, which transmit this rhythmic input to mitral and tufted cells of the OB. *Via* the olfactory cortex, RR reaches the neocortex and subcortical structures. In a presumed corollary discharge pathway, brain stem signals might in addition directly impose RR on cortical circuits (black).

Mitral and tufted cells in the OB synapse on neurons in the piriform cortex and other olfactory areas such as the anterior olfactory nucleus and entorhinal cortex (see Lane et al., 2020 for a review on olfactory sensory pathway), which connect to the neocortex. RR activity thus reaches the neocortex and hippocampus in a multi-synaptic pathway (Canning et al., 2000) and eventually gives rise to respiration-synchronized LFP oscillations, although the quantitative contributions of different anatomical routes remain to be established (Figure 2). There is indeed a causal relationship between nasal airflow, the OSN-OB system, and the occurrence of central RR: Tracheotomy, naris occlusion, chemical lesioning of OSNs with methimazole as well as removal or inactivation of the OB consistently eliminate RR oscillations in the neocortex and hippocampus (Ito et al., 2014; Yanovsky et al., 2014; Biskamp et al., 2017; Moberly et al., 2018). In addition, respiration-synchronous cortical oscillations are absent when cats use oral instead of nasal breathing (Cavelli et al., 2020). Three lines of evidence support the notion that RR in the cortex reflects a “true” local signal rather than being volume-conducted from the OB. First, local neurons are modulated by ongoing RR. Both neocortical and hippocampal neurons discharge phase-coupled to RR (Yanovsky et al., 2014; Biskamp et al., 2017; Karalis and Sirota, 2018; Moberly et al., 2018; Bagur et al., 2021), and whole-cell recordings indicated respiration-synchronous subthreshold membrane oscillations in pyramidal neurons, initially in the piriform cortex (Fontanini et al., 2003) and more recently in the parietal cortex (Jung et al., 2019). Notably, these subthreshold oscillations are reduced when airflow through the nose is prevented by tracheotomy (Fontanini et al., 2003), further supporting the causal role of nasal airflow for cortical RRs. Second, current source density analysis identified respiration-synchronous current sinks in deep layers of the mPFC and in the hippocampal dentate gyrus (Lockmann et al., 2016; Karalis et al., 2016). Third, consistent with this result, Bagur et al. (2021) showed stronger coherence with the OB signal in deep than superficial layers of the mPFC.

While the origin and local nature of cortical RR LFP oscillations are thus well-validated, two key questions remain open: First, it is not entirely clear how the large-amplitude respiration-synchronous LFP is generated in the hippocampus and neocortex, or in other words how rhythmically oscillating discharges in axons from olfactory regions are translated into rhythmically active local populations (“translation problem”). A series of findings emphasize a central role of GABAergic interneurons in the generation of local RR: Single-unit recordings showed that a high proportion of GABAergic cells is phase-entrained by RR oscillations, and/or directly by rhythmic breathing, while the proportion of significantly coupled pyramidal cells is lower (Biskamp et al., 2017; Karalis and Sirota, 2018; Kőszeghy et al., 2018; Folschweiller and Sauer, 2021). Furthermore, subthreshold membrane oscillations in pyramidal cells of the parietal cortex seem to reflect inhibitory inputs, as they increase in amplitude when the holding potential is moved away from the reversal potential of GABAergic currents (Jung et al., 2019). It is thus possible that RR primarily relies on local interneurons, which might be rhythmically biased in their firing by long-range input from olfactory regions and impose the RR on the local pyramidal cell population. Silencing experiments and *in vivo* patch-clamp recordings from local interneurons should shed further light on that hypothesis in the future. Additionally, the neocortex of rodents responds best to specific frequencies of the respiration, with a peak correlation at 1.6 Hz in the barrel cortex of anesthetized animals (Ito et al., 2014), and a non linear entrainment of the mPFC LFP by optogenetic stimulation of the OB at a range of frequencies in sleeping mice (Bagur et al., 2021), suggesting that the resonance properties of the local cortical network might aid the broadcasting of the RR.

Second, it remains controversial to what extent nasal airflow-driven feedback contributes to the entrainment of cortical neurons. Ablation of OSNs by systemic application of methimazole (Bergman et al., 2002) has been reported to efficiently eliminate slow RR and RR-entrained fast gamma activities in the prefrontal cortex and hippocampus, but only partially affected the entrainment of local neurons to ongoing rhythmic breathing (Karalis and Sirota, 2018; Moberly et al., 2018). Karalis and Sirota (2018) suggested a corollary discharge or efference copy mechanism (Sperry, 1950; von Holst and Mittelstaedt, 1950) that transmits respiration-synchronous activity indirectly from the brainstem rhythm generator to the cortex (Figure 2). This mechanism might inform cortical circuits about the body movement caused by the act of breathing to adjust proprioception accordingly. However, the anatomical underpinnings of the proposed corollary discharge mechanism are to date unknown. Neurons of the pre-Bötzinger complex, the primary site of generation of the RR, do not directly project to the neocortex (Yang and Feldman, 2017). Connections from pre-Bötzinger complex to the thalamus (Yang and Feldman, 2017), in particular the mediodorsal nucleus, and to the locus coeruleus (Yackle et al., 2017), which both project onwards to the forebrain, have been suggested as a potential anatomical substrate (Karalis and Sirota, 2018). Future work is needed to clarify the quantitative contribution of the presumed corollary discharge-induced entrainment of local circuits, ideally with methods that reversibly rather than chronically inactivate the OSN-OB-neocortex pathway.

## The Role of Respiration Driven-Oscillations in Emotional and Cognitive Circuits

The widespread occurrence of RR across the brain suggests an important role in the modulation of emotion and cognition. The breathing rhythm changes in frequency, regularity, and amplitude depending on emotional and arousal states, thus making it adapted to support cognition in different ways depending on the emotional context. Another hint of the importance of respiration in the forebrain is the highly conserved direct pathway to cortical areas of the olfactory system which, unlike other senses, bypasses the thalamus and is intricately connected with the limbic system. In the following segments, we will describe potential functions of RR for distinct states of emotional cognition.

### The Role of RR in the Modulation of Fear Behavior

A particularly well-validated role of the RR in emotional cognition is its involvement in the regulation of fear behavior.

**Figure 3:**
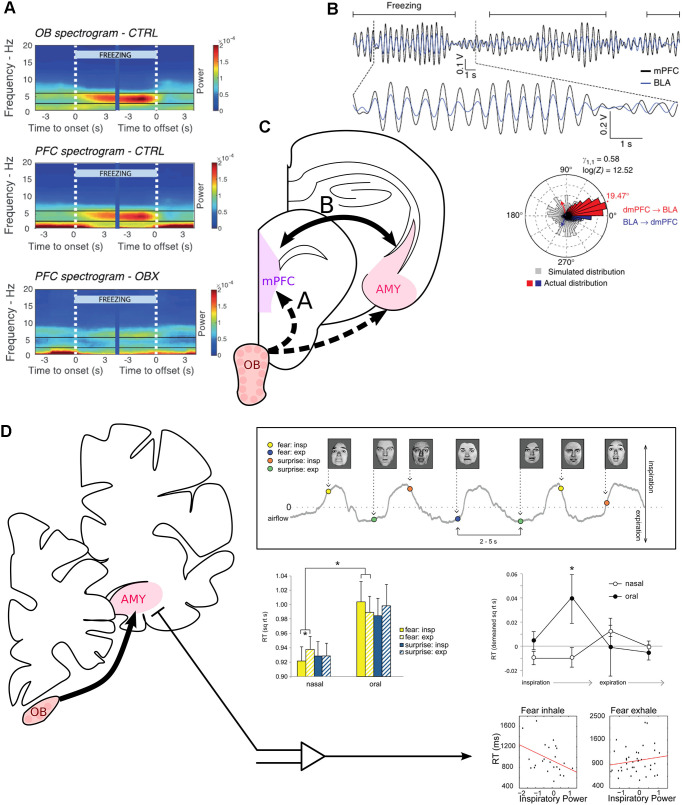
Modulation of fear-related neural correlates and recognition of fearful faces by respiration. **(A)** The mPFC is entrained at 4 Hz by RR sensorial afferences during freezing. Spectrogram averaged across mice at freezing onset and offset, indicated by the white vertical dashed lines. Power spectra of the OB (top) and medial prefrontal cortex (PFC, middle) of control mice. Bottom: Power spectrum of the PFC of mice after bulbectomy. Reprinted from Bagur et al. (2021) under CC BY 4.0. **(B)** The mPFC and the basolateral amygdala (BLA) are highly coherent at 4 Hz during freezing. Top: Bandpass filtered 2–6 Hz LFP signals of the mPFC (black) and the simultaneously recorded BLA LFP (blue) during recall of conditioned fear. Freezing epochs are indicated by the horizontal black lines. Bottom: Phase difference between the mPFC and the amygdala (Red: above 0° of phase difference, mPFC phase precedes BLA phase. Blue: Below 360°, BLA phase precedes mPFC phase. Gray: simulated phase differences obtained by bootstrapping). Adapted by permission from Springer Nature, Nature Neuroscience, Karalis et al. (2016), copyright (2016). **(C)** The periamygdaloid cortex receives direct projection from the OB. BLA units are furthermore paced by RR in the home cage (Karalis and Sirota, 2018). **(D)** Inspiration facilitates the recognition of fearful faces. Top right, experimental paradigm: subjects had to discriminate between fearful or surprised faces presented at jittering interval of 2–5 s during nasal or oral breathing (gray line). Middle left: reaction time to recognize expressions for each emotion (yellow: fear, blue: surprise), depending on the phase of respiration (full: inspiration, dashed: expiration) and the breathing route. Middle right: Detrended reaction time for the recognition of fear for four phases of the breathing cycle. Oral breathing induced an increase in the reaction time during late inspiration. Bottom: Correlation between reaction time and the mean z-scored power in the delta band during the whole inspiration or expiration time window. On the left, “Fear inhale” shows the results for fearful faces presented during inspiration, and “Fear exhale” the results for fearful faces presented during expiration. Reprinted from Zelano et al. (2016) under CC BY 4.0. *p < 0.05.

During auditory fear conditioning-induced freezing, the mPFC LFP and single units are strongly paced at the very regular 4 Hz frequency of the RR that mice exhibit in that state (Figure 3A; Karalis and Sirota, 2018; Moberly et al., 2018; Bagur et al., 2021), which seems to be the optimal breathing pattern to entrain cortical areas (Girin et al., 2021). Disrupting the olfactory sensorial afferences has been reported to increase the level of freezing (Moberly et al., 2018), to not affect freezing (Karalis and Sirota, 2018), or to decrease the length of freezing bouts, in the latter case highlighting a role of the RR in the maintenance of freezing (Bagur et al., 2021). These contradicting results on whether the RR supports or opposes freezing could be imputed to the fact that the mPFC, which is strongly entrained by RR during that behavior, can have bidirectional effects on the level of freezing (Sierra-Mercado et al., 2011). In addition, technical aspects might play a role: Bagur et al. (2021) recently showed that systemic application of methimazole, which was used to ablate OSNs in two studies reporting enhanced or unaltered levels of freezing (Moberly et al., 2018; Karalis and Sirota, 2018), results in enhanced freezing-like states in the absence of aversive foot shocks. These data suggest that unspecific behavioral alterations might confound the interpretation of OSN ablation on fear memory using this approach. In support of a role of the RR in the maintenance of freezing, previous work on putative RR in the mPFC, i.e., a 4 Hz rhythm entraining the mPFC LFP during freezing, showed that closed-loop optogenetic inhibition of the mPFC “in-phase” of the local 4 Hz rhythm disrupted freezing, while inhibition out of phase had the opposite effect (Dejean et al., 2016). Another study of the putative RR suggests it has a role in information flow from the mPFC to the basolateral amygdala (BLA; Figure 3B; Karalis et al., 2016). Interestingly, the part of the amygdala involved in conditioned fear, the BLA, and the PFC, do not receive direct projections from the OB. Nonetheless, the mPFC is likely entrained by the OB during freezing by a multisynaptic pathway (Figure 3A; Moberly et al., 2018; Bagur et al., 2021) as retrograde tracing from the mPFC did not reveal any direct projections from the OB, but optogenetic stimulation of the OB at different frequencies did result in entrainment of the mPFC LFP in a non-linear manner, implying an active propagation mechanism. So far, no simultaneous recording of the BLA LFP and respiration during freezing has been reported, but the respiration-paced BLA activity under neutral conditions (i.e., in the animal’s home cage; Figure 3C; Karalis and Sirota, 2018) together with the high coherence with the mPFC LFP at 4 Hz during freezing strongly imply that the BLA is entrained by the RR during freezing. Altogether, these studies suggest a role of the RR in mediating communication between the mPFC and the BLA to control fear behavior.

The amygdala can be divided into several sub-regions with different connectivity and behavioral correlate. While the BLA, as mentioned above, does not receive direct input from the OB, the cortical amygdala (CoA) and the medial amygdala (MeA) are directly targeted by the main OB and involved in odor induced innate fear (Root et al., 2014; Isosaka et al., 2015). The projections from the main OB to the amygdala thus constitute a potential direct entry point for the RR to pace neuronal activity related to fear expression. Interestingly, the mPFC is also entrained by the RR during innate fear induced with odorants (Karalis and Sirota, 2018), and the IL cortex, which has the strongest entrainment by the RR, has direct projections to the CoA and MeA (Mcdonald et al., 1996). One could speculate that the IL synchronizes its activity *via* the RR to regulate the activity of the amygdala in innate fear behaviors in rodents as well.

So far, most of the studies about the role of the RR in fear were conducted on rodents, but the few experiments conducted on other species suggest that the entrainment of the amygdala by the RR is highly conserved in mammals. In cats, approximately 10% of the central amygdala cells fire respiration-synchronized in a neutral state (Zhang et al., 1986). In humans, the direct projections from the OB to the MeA and the CoA are also conserved (Lane et al., 2020), and faster brain oscillations have been shown to be phase-locked with the respiration in the amygdala during anticipation of an electric shock (Masaoka and Homma, 2000). Furthermore, it seems that breathing with a pattern characteristic of fear can generate feelings of fear, anxiety, and anger (Philippot et al., 2002), but it is still not known whether the activation of the direct projection from the OB to the amygdala, by odorants or airflow, is sufficient to trigger fear in humans. Nonetheless, sensorial reafferences coming from nasal breathing do influence another fear-related cognitive process, which is fear recognition.

### The Role of the RR in Fear Recognition

In the amygdala, different sub-bands of the LFP, including delta, appear to be phase lock with inspiration during nasal breathing in humans (Zelano et al., 2016). Odors still play a facilitating role in visual fear recognition in humans (Kamiloglu et al., 2018), perhaps *via* direct projection from the OB onto the periamygdaloid cortex (Lane et al., 2020), which is involved in the recognition of fearful faces (Morris et al., 1996). But more interestingly, even in the absence of fear-related odorants, breathing in through the nose was also found to facilitate the recognition of fearful but not surprised faces (Figure 3D; Zelano et al., 2016). In one subject from whom the authors measured the amygdala LFP during the task, they observed that the patient was faster at the recognition of fear when the amygdala delta inspiratory power was higher. Importantly, the work of Zelano et al. (2016) shows that the RR influences the recognition of fear in humans in the absence of specific smells, potentially *via* an inspiration-triggered resetting of the local oscillations.

Overall, the sensorial afferences coming from the olfactory system can modulate fear-related behavior and cognition without carrying olfactory components, suggesting that the RR plays a role in different fear-related processes, potentially *via* the projection from the OB to the amygdala.

### Potential Implications of RR in Other Emotional States

#### Despair

Besides fear, another strong negative emotional state paired with high arousal during which respiration seems to play a key role is despair-like behavioral responses to an unavoidable acute stress. Indeed, a very similar 4 Hz RR as during freezing recruits the mPFC LFP and single units during immobility in a tail suspension test (Figure 1D; Biskamp et al., 2017). As for freezing, the mPFC can also bidirectionally influence the response to challenging situations by promoting despair-like behavior or struggle (Warden et al., 2012), but the exact role of the RR in this behavior requires further investigation. Interestingly, bulbectomized rodents reliably show depressive-like behavior, including enhanced immobility during tail suspension (Song and Leonard, 2005). However, behavioral symptoms in bulbectomized animals start only around 2 weeks after bulbar ablation, arguing against an immediate effect of the loss of RR and/or olfactory inputs on the development of depressive symptoms. The congenital lack of olfactory sensorial afferences furthermore increases the prevalence of depression in humans (Croy et al., 2012). In addition, in the case of patients with chronic breathing disorders, where the RR is qualitatively disrupted, up to 80% of the patients show depression or anxiety disorders (Kunik et al., 2005). Unfortunately, it is currently not possible to deduce how much the reduced RR in the limbic network and the impaired olfactory inputs contribute to depressive symptoms in chronic breathing disorders and congenital anosmia.

#### Reward and Motivation

Recent publications have identified RR in several brain regions involved in reward, motivation, and addiction. One study described the RR entrainment of neurons in the ventral striatum (Karalis and Sirota, 2018). The ventral striatum is composed of the nucleus accumbens (NAcc) and the striatal part of the olfactory tubercle (OT). The OT is a direct target of the OB in rodents, monkey, and humans (Carmichael et al., 1994; Lane et al., 2020), thus providing a potential anatomical basis for the emergence of RR in the striatum. On the behavioral level, it has been shown that cocaine infusion in the OT but not in the NAcc nor the ventral pallidum induces place preference (Ikemoto, 2003). Moreover, rats learn to self-inject cocaine in their OT and NAcc shell, which is the dorsal extension of the OT, but not in the NAcc core, ventral pallidum, or dorsal striatum (Ikemoto, 2003). These results suggest that striatal regions with direct input from the OB are particularly important for the rewarding effects of the drug. It should be noted that the author observed a stronger rewarding effect in the medial OT, which is also receiving sparser inputs from the OB (Wesson and Wilson, 2011), suggesting a gradient between a lateral OT more involved in smell processing and a medial OT involved in reward. The OT sends projections to the orbitofrontal cortex, a prefrontal cortex area in which nearly all recorded single units are entrained by the RR in head-fixed animals (Kőszeghy et al., 2018), and which is thought to encode (among other things) reward expectation (Noonan et al., 2012).

**Figure 4:**
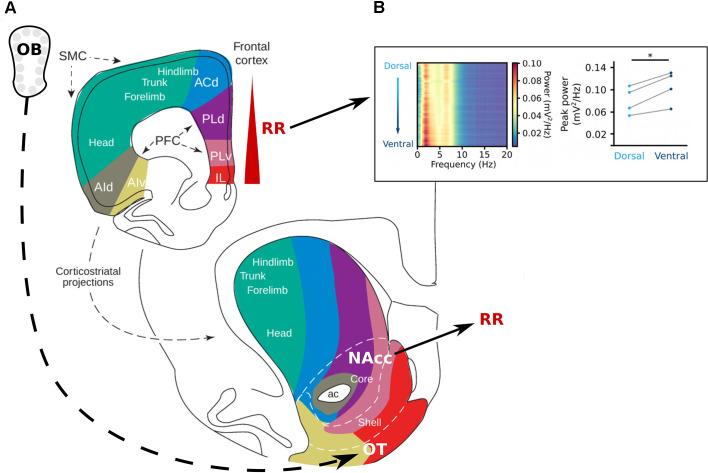
The reward system and respiration-related oscillations. **(A)** Topography of the projections from the frontal cortex to the striatum. The olfactory tubercle (OT) receives direct projections from the OB and the nucleus accumbens (NAcc) has been shown to be strongly entrained by RR (Karalis and Sirota, 2018). In the prefrontal cortex, the RR power increases from dorsal to ventral (Karalis and Sirota, 2018; Folschweiller and Sauer, 2021). Adapted from Voorn et al. (2004), copyright (2004) Trends in Neurosciences, with permission from Elsevier. **(B)** Left: Power spectra of the mPFC along the dorsoventral axis, shows an increase in power at the frequency of respiration in the ventral direction. Left: Peak power for each mouse at 4 Hz at the most superficial recording site (light blue) and the deepest recording site (dark blue) of the mPFC. Reprinted from Folschweiller and Sauer (2021) under CC BY 4.0. *p < 0.05.

There is additional evidence that neocortical inputs to the ventral striatum are more strongly paced by RR than inputs to the dorsal striatum. Karalis and Sirota (2018) observed a gradual decrease of the RR entrainment of the mPFC from ventral to dorsal. There is a medioventral to laterodorsal topography of connections between the mPFC and the striatum, with the IL being more connected to the OT, the PrL to the NAcc, and the ACC and motor cortices to the dorsal striatum (Figure 4; Voorn et al., 2004; Gabbott et al., 2005). These results demonstrate that interconnected brain regions involved in processing rewarding stimuli are paced by RR, raising the possibility that RR entrainment might support the encoding of reward-related information by synchronizing distant brain regions, similar to as it has been suggested for the coordination of mPFC and amygdala during the recall of fear memory. However, causal tests of such a potential function of RR remain to be performed.

To sum up, the RR rhythm has been recorded in multiple areas involved in emotion and higher-order cognitive functions in both rodents and humans. Behavioral performance is modulated by respiration (Zelano et al., 2016; Moberly et al., 2018; Bagur et al., 2021). Therefore, the role of RR in the brain is clearly broader than the support of olfactory processing. But it is important to note that the basic behaviors observed are not fully disrupted when the olfactory sensorial afferences are removed and the RR rhythm can have ambivalent effects on emotional cognition, possibly pointing toward a role in cognitive flexibility and emotion monitoring. In rodents, the RR oscillations might fulfill this role by synchronizing distant brain regions, neocortical areas such as the mPFC, with more ventral nuclei and paleocortices such as the amygdala and the OT that are strongly connected to the olfactory system. Additionally, the processing of smells is tightly linked to the RR, as well as whisking (Figure 1A; Ito et al., 2014), and respiration has been argued to be an important synchronization signal for sensorimotor integration (for review see Kleinfeld et al., 2014).

## Mechanisms of How Breathing Might Impact Cognition

### Modulation of Gamma Oscillations

Brain activity is characterized by a vast family of oscillation patterns (see Buzsáki and Draguhn, 2004 for a review on different oscillations). Respiration might affect local circuit computation by modulating gamma oscillations (30–100 Hz). Gamma activities are strongly associated with cognitive functions: As mice perform a delayed non-match-to-place paradigm in a T-maze, hippocampal high-frequency gamma oscillations occur specifically during the decision phase as the mice approach the choice point in the maze (Yamamoto et al., 2014). Moreover, gamma oscillations are impaired in psychiatric disorders which present with cognitive disturbances (for review see Uhlhaas and Singer, 2010). Gamma oscillations occur nested in ongoing theta activities (for review see Colgin, 2015). This temporal organization of gamma oscillations by theta seems crucial for the behavioral functions. Tort et al. (2009) showed that theta-gamma coherence increased during learning of an item-context matching task. Similarly, another study demonstrated increasing 20–40 Hz gamma-theta coherence during learning of an odor-place association task (Igarashi et al., 2014). Furthermore, gamma oscillations in the neocortex are synchronized to hippocampal theta, suggesting that theta phase coupling might provide a general mechanism of information transfer across regions (Sirota et al., 2008).

**Figure 5:**
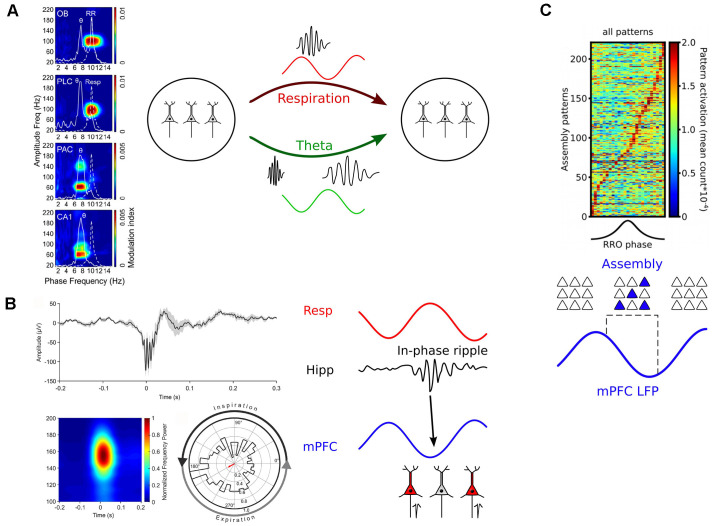
Mechanisms of how breathing might impact cognition. **(A)** Left: RR entrains fast gamma activities across brain circuits. PLC, prelimbic cortex; PAC, parietal cortex. Reprinted from Zhong et al. (2017). Right: Respiration and theta oscillations provide timing signals for gamma oscillations of different frequencies (Biskamp et al., 2017; Zhong et al., 2017). **(B)** Left: Respiration paces ripples. Top: Example of a hippocampal ripple. Bottom: Average ripple power spectrum (left) and distribution of ripple events as a function of respiration phase. Reprinted from Liu et al. (2017) under CC BY 4.0. Right: RR-entrained ripples might enable efficient communication between hippocampal and neocortical circuits (Liu et al., 2017; Karalis and Sirota, 2018). **(C)** Top: Neuronal assemblies activate during the descending part of the prefrontal respiration-related oscillation (RRO). Reprinted from Folschweiller and Sauer (2021) under CC BY 4.0. Bottom: RR creates time windows for assembly activation and might thus orchestrate the joint activity of distributed assembly members or facilitate assembly stabilization by offline reactivation (Dejean et al., 2016; Folschweiller and Sauer, 2021).

Interestingly, recent work demonstrated that respiration-driven neocortical and hippocampal oscillations pace gamma activities. In the prefrontal cortex of mice, high-frequency gamma (80–100 Hz) is strongly entrained by RR (Biskamp et al., 2017; Zhong et al., 2017; Karalis and Sirota, 2018), while slow gamma (~40–80 Hz) and very high gamma (130–150 Hz) are entrained by theta oscillations (Biskamp et al., 2017; Zhong et al., 2017). This observation led to the hypothesis that theta and RR constitute two different channels of communication coupling to different gamma sub-bands (Figure 5A). In addition, gamma activities are synchronized with respiration across OB and wide areas of neocortex in the awake cats (Cavelli et al., 2020), suggesting that these principles apply to distinct species (it should be noted that 40 Hz gamma was entrained in cats, pointing to potential species-specific differences in modulated frequency that will require further investigation). Finally, recordings from the barrel cortex exhibited cross-frequency coupling between respiration and ~75 Hz gamma (Ito et al., 2014). These findings are supported by a different study reporting phase-coupling between regular ~3 Hz OB potentials and high gamma activity in the hippocampus, motor, and sensory cortex (Rojas-Líbano et al., 2018), suggesting that the gamma-pacing effect of respiration might be a common principle across brain circuits.

It is likely that RR entrains hippocampal gamma oscillations *via* the entorhinal cortex (Nguyen Chi et al., 2016; Karalis and Sirota, 2018), which receives direct afferents from the OB (in monkeys, only a small rostral part of the entorhinal cortex receives direct inputs from the OB; Carmichael et al., 1994, which is also likely the case in humans; Insausti et al., 2002). RR synchronization of synaptic inputs from the medial entorhinal cortex (MEC) persists after olfactory deafferentiation, while respiration-rhythmic inputs from the lateral entorhinal cortex (LEC) are gone (Karalis and Sirota, 2018). The MEC has been argued to be important for “internal navigation” (using path integration), while the LEC might have a more prominent role in “external navigation” (using landmarks/cues; Connor and Knierim, 2017), suggesting that respiration might differentially affect afferents carrying distinct qualities of spatial information. Thus, the human hippocampus, in which delta oscillations are phase-locked to inspiration and depend on nasal breathing (Zelano et al., 2016), could be entrained by the means of sensorial afferences transmitted by the entorhinal cortex. On the behavioral level, it has indeed been demonstrated that encoding, consolidation, and recall of episodic memory can be modulated by respiration in humans (Zelano et al., 2016; Arshamian et al., 2018; Nakamura et al., 2018), with the discrete phase of the respiration affecting performance during encoding and recall, while the constant rhythmic airflow through the nasal cavity is relevant for consolidation. Importantly, experimentally disrupting the gamma range synchronization between the entorhinal cortex and the hippocampus impairs learning of spatial tasks in rodents (Fernández-Ruiz et al., 2021). Based on these results, it is possible that RR-nested gamma activities that are driven by entorhinal input support memory formation in the hippocampus.

Respiration might further impact gamma oscillations during sleep. In rapid eye movement (REM) sleep, during which theta-nested gamma oscillations are prevalent, the slow RR is found in the neocortex, with larger power in rostral areas such as the anterior cingulate cortex (Tort et al., 2018). RR frequency modulates the coupling between theta and gamma oscillations in the parietal cortex during REM sleep (Hammer et al., 2021). Changes in respiration frequency precede changes in slow gamma in the parietal cortex, but changes in parietal theta activity precede changes in breathing, suggesting a feedback loop interaction: The level of arousal during REM (equivalent to theta power) might change the breathing pattern, which in turn would modulate the parietal slow gamma oscillations (Tort et al., 2021). Taken together, these results emphasize multiple levels of control over gamma activities by respiration: Much like theta oscillations, RR defines time windows of preferred gamma activity in awake mice. Moreover, respiration controls theta-nested gamma oscillations during sleep.

### Modulation of Ripples

Ripple oscillations are short bouts of high-frequency activity in CA1, which occur during offline states (sleep, immobility) and reflect highly synchronized firing of CA1 pyramidal neurons (O’Keefe, 1976, see Buzsáki, 2015 for review). Importantly, neuronal sequences related to visited places as well as upcoming trajectories are re- and pre-played, respectively, on compressed timescales during ripples (Lee and Wilson, 2002; Foster and Wilson, 2006; Diba and Buzsáki, 2007). These observations gave rise to the hypothesis that replay/preplay during ripples relates to the consolidation of learned behavioral sequences and the planning of future trajectories (Buzsáki, 2015). Experimentally suppressing ripples in rats during post-learning sleep indeed impaired learning in a spatial task, underscoring the relevance or ripples for memory formation (Girardeau et al., 2009). Moreover, ripples occur synchronously in higher-order neocortical areas and hippocampus in a learning-dependent manner, suggesting that they might aid the transfer of memory content to the neocortex (Khodagholy et al., 2017).

During awake immobility, when ripple frequency is high, RR seems ubiquitous in the forebrain. In mice, the RR occurs prominently in the mPFC, orbitofrontal cortex, and in the parietal cortex and hippocampus (Zhong et al., 2017; Kőszeghy et al., 2018). In humans, there is a global entrainment of neuronal activity at rest (Herrero et al., 2018; Kluger and Gross, 2020). It is worth noting that most of the areas entrained by respiration in humans belong to the default mode network (posterior cingulate cortex, angular gyrus, and the precuneus), the dorsal attention network (frontal eye fields, posterior and anterior intraparietal sulcus), and the salience network (anterior cingulate cortex, ventrolateral prefrontal cortex, and insula; Kluger and Gross, 2020). Interestingly, in recent work Liu et al. (2017) directly demonstrated that hippocampal ripples are entrained by respiration in rodents: They preferentially occur during early expiration (Figure 5B). Chemogenetically suppressing OB activity disrupted the respiration phase-locking of hippocampal ripples. In line with this observation, a different study found CA1 ripples to occur predominantly during the post-inspiratory phase (Karalis and Sirota, 2018). The synchronous activity of mPFC and NAcc neurons during hippocampal ripples was more frequent for ripples happening in their preferred phase of respiration. However, in contrast to the study by Liu et al. (2017), ablating OSNs with methimazole did not affect ripple entrainment, suggesting that a corollary discharge rather than a peripheral feedback mechanism *via* the OSN-OB route might underlie ripple entrainment. Despite different mechanistic results, these studies suggest that ongoing respiration-driven oscillations contribute to the organization of ripple activity, and might thus provide a causal link to memory consolidation.

### Modulation of Neuronal Assemblies

The building blocks of cortical computations are thought to be comprised of groups of coactive cells, called neuronal assemblies, rather than single neurons (Buzsáki, 2010; Papadimitriou et al., 2020; El-Gaby et al., 2021). Assembly neurons become active together and are presumed to efficiently impact downstream readers due to their synchronized activation (Buzsáki, 2010). Given that respiration provides windows of preferred neuronal activity, it would be conceivable that respiration might directly regulate when neuronal assemblies activate. In recent years, it has become possible to reliably extract assembly activations from electrophysiological data using dimensionality reduction and strict statistical methods that allow the detection of transient and repeated neuronal coactivity (for review see Lopes-dos-Santos et al., 2013). In a pioneering study, Dejean et al. (2016) established a link between neuronal assembly activation and the expression of fear memory in mice. They showed that freezing responses of mice are accompanied by the emergence of assembly activations specifically in the ascending phase of ongoing 4 Hz oscillations. Although not directly demonstrated in that study, several laboratories have since shown that freezing-related 4 Hz activities are respiration-driven oscillations (Karalis and Sirota, 2018; Moberly et al., 2018; Bagur et al., 2021). Interestingly, optogenetic manipulation of fear-related assemblies in a 4 Hz phase-specific manner could bi-directionally modulate the amount of freezing, suggesting a causal role of assembly activity nested in respiration-driven prefrontal oscillations (Dejean et al., 2016). More recently, recordings from head-fixed mice presented evidence that the modulation of assembly patterns by RR might generalize to behavioral states other than fear (Figure 5C; Folschweiller and Sauer, 2021). In the absence of fearful stimuli, prefrontal assembly patterns are preferentially activated during the descending phase of respiration-driven oscillations. These data jointly show that respiration provides windows of opportunity for assembly activation, and that distinct phases of respiration might be linked with distinct cognitive/emotional content (e.g., freezing responses during the ascending phase vs. default or neutral state during the descending phase). On the mechanistic level, phase-specific pooling of assembly activity could support the brain-wide synchronization of distributed assembly members (Karalis et al., 2016), or facilitate spontaneous reactivation of assemblies as proposed by theoretical accounts to protect assemblies against degradation by synapse turnover (Fauth and van Rossum, 2019).

## Conclusions and Outlook

There is a clear reciprocal relationship between emotions and respiration: while emotion and cognition modify the respiratory pattern to best fit the context-dependent bodily needs for oxygen, RRs entrain brain circuits to support neuronal computation. RRs come about *via* sensorial afferences from the OB, which are paced by the airflow in the nasal cavity and target multiple brain regions along the rostro-caudal axis. While the exact pathway(s) from the OB to associative areas is still unclear, there is evidence of the involvement of interneurons in the broadcasting of this slow oscillation in the neocortex. The ubiquitous feature of the RR is highly conserved across species and it has been recorded widely across the brain in a large variety of emotional and arousal states. Finally, the modulation of faster brain oscillations and transient synchronization of neurons as assemblies shows that the RR plays a role at the core of neuronal processing.

While tremendous progress in the field of respiration-driven pacing of brain circuits has been made in recent years, we are still far from having a clear picture of the mechanisms by which respiration impacts cognitive functions. We propose the following key challenges to be addressed in future experiments to close that gap in our understanding:

(1) Tackling the translation problem:

It will be crucial to understand how respiration-synchronous activities are converted to local respiration-rhythmic oscillations in the neocortex. Inspiration might be drawn from well-studied oscillation species such as gamma activities, which rely on parvalbumin-positive interneurons (Sohal et al., 2009; for review see Hu et al., 2014). Recordings from identified interneuron types, preferably with access to subthreshold membrane oscillations *via* whole-cell recording or voltage-sensitive dye imaging might be a preferred experimental strategy to shed light on the contribution of different circuit components to the translation of respiration-driven signals. Furthermore, monitoring which inter-areal axonal projections show respiration-paced activities might help in guiding toward a more complete understanding of how respiration-driven patterns are distributed across the neocortex.

(2) Dissecting external feedback vs. corollary discharge:

We require more knowledge about the contribution of the OSN-OB pathway and the presumed direct efference copy from the brain stem to local effects of respiration. Reversible silencing of both pathways combined with unit recording during behavior might be the ideal way. However, while the OSN-OB pathway has been successfully manipulated using optogenetics (Bagur et al., 2021), the complex anatomy of the brain stem, the so far unclear route(s) of the presumed corollary discharge mechanism to the neocortex, and the vital function of many brain stem center render optogenetic silencing experiments during behavior difficult. An alternative approch could be to study the impact of external feedback vs. corollary discharge on local unit activity in species capable of natural mouth breathing (e.g., cats, Cavelli et al., 2020).

(3) Understanding respiration-dependent synchronization on mesoscopic scales:

Coherent respiration-synchronous oscillations among different brain regions have been described but have so far been limited to few regions and/or behavioral states (e.g., synchronous 4 Hz activity between mPFC and amygdala during fear memory; Karalis et al., 2016). It would be appreciable to reveal inter-regional synchronization between multiple brain areas (e.g., across the neocortex) and across behaviors (e.g., neutral, aversive, and appetitive states). Multi-region electrophysiological recording techniques (Khodagholy et al., 2017) or whole-hemisphere voltage-sensitive dye imaging could prove useful for such endeavors.

(4) Identifying universal functional principles.

Respiration-paced activities occur across species. However, respiration frequency differs by an order of magnitude between mice (~2–5 Hz in rest) and humans (~0.2–0.3 Hz in rest), while the frequencies of faster brain oscillations and action potential kinetics are largely preserved (Buzsáki et al., 2013). This raises the question to what extend the pacing of brain circuits by respiration might follow common principles, and which mechanisms might be species-specific.

## Author Contributions

SF and J-FS performed literature research and wrote the manuscript. All authors contribute to the article and approved the submitted version.

## Conflict of Interest

The authors declare that the research was conducted in the absence of any commercial or financial relationships that could be construed as a potential conflict of interest.

## Publisher’s Note

All claims expressed in this article are solely those of the authors and do not necessarily represent those of their affiliated organizations, or those of the publisher, the editors and the reviewers. Any product that may be evaluated in this article, or claim that may be made by its manufacturer, is not guaranteed or endorsed by the publisher.
